# Light-Dependent Effects of Prefrontal rTMS on Emotional Working Memory

**DOI:** 10.3390/brainsci11040446

**Published:** 2021-03-31

**Authors:** Anne Weigand, Lisa Edelkraut, Markus Conrad, Simone Grimm, Malek Bajbouj

**Affiliations:** 1 MSB Medical School Berlin, Rüdesheimer Straße 50, 14197 Berlin, Germany; simone.grimm@medicalschool-berlin.de; 2 Department of Psychobiology and Methodology of Behavioral Sciences, Faculty of Psychology, University of Málaga, Av. de Cervantes, 2, 29016 Málaga, Spain; lisa.edelkraut@gmail.com; 3 Unit of Cognitive Neurology and Aphasia (UNCA), Centro de Investigaciones Médico–Sanitarias (CIMES), University of Malaga, C/ Marqués de Beccaria, 3, 29010 Málaga, Spain; 4 Department of Cognitive, Social and Organizational Psychology, Universidad de La Laguna, Campus Guajara, s/n, 38071 San Cristóbal de La Laguna, Spain; maconrad@ull.es; 5 Department of Psychiatry and Psychotherapy, Campus Benjamin Franklin, Charité—Universitätsmedizin Berlin, Hindenburgdamm 30, 12203 Berlin, Germany; malek.bajbouj@charite.de

**Keywords:** emotion, working memory, state-dependency, colored light exposure, transcranial magnetic stimulation

## Abstract

Growing evidence suggests that colored light exposure can affect several brain functions in addition to conscious visual perception. Blue as compared to green light has especially been shown to enhance alertness and vigilance, as well as cognitive functions. However, the role of light exposure in studies using non-invasive brain stimulation remains unclear. Here, we examined the impact of light on cognitive-emotional effects of prefrontal repetitive transcranial magnetic stimulation (rTMS). In a randomized within-subjects design, twenty participants (12 males, 26 ± 4 years) were exposed to blue or green light prior and concomitant to active or sham rTMS (1Hz, 15min, 110% of the resting motor threshold), applied over the right dorsolateral prefrontal cortex (DLPFC). In each condition, an emotional working memory task (EMOBACK) was presented pre- and post-intervention. Stimuli of the EMOBACK task were positive, negative and neutral words. Our results revealed valence-specific stimulation effects in dependence of colored light exposure. More specifically, task accuracy was significantly increased for positive stimuli under blue light and for negative stimuli under green light exposure. Our findings highlight the importance of state-dependency in studies using non-invasive brain stimulation and show blue light exposure to be a potential adjunctive technique to rTMS for enhancing cognitive-emotional modulation.

## 1. Introduction

Light is not only necessary for vision but also exerts a wide range of effects on other brain functions, including cognitive and emotional processing [1,2,3,4,5,6]. These non-visual effects of light are mediated by a specific type of photoreceptor, the intrinsically photosensitive retinal ganglion cells (ipRGCs) [7]. While the maximum sensitivity of the ipRGCs lies in the blue short wavelengths of the light spectrum, the classic photoreceptors responsible for vision are maximally sensitive to longer wavelengths such as green light [1,2,3,5]. Several studies show that the spectral quality of blue light increases the cognitive functions [8,9,10] as well as brain activity involved in working memory tasks [11,12,13] and in response to emotional stimuli [3]. For example, during an auditory working memory task, less than 60 seconds of blue light exposure prompts the activation of supplemental prefrontal and thalamic brain regions associated with alertness and cognition as well as primary hubs of the default mode network [14]. Further to this, blue light exposure significantly improved subjectively [6,9,10] and objectively [15] measured alertness and vigilance, and accelerated relaxation processes after induced stress [16]. In contrast, green light has been shown to reduce arousal responses [1,5] and to decrease brain activity during working memory tasks [13].

Both cognitive and emotional effects have been extensively studied using non-invasive brain stimulation, such as repetitive Transcranial Magnetic Stimulation (rTMS) [17,18,19]. For example, applying rTMS to the dorsolateral prefrontal cortex (DLPFC) has been shown to modulate functions such as alertness [20], cognitive control [21], emotional processing [22] as well as working memory [23]. In our own previous work [24,25], we applied offline rTMS to the DLPFC prior to a newly developed emotional working memory task (EMOBACK). The EMOBACK task allows investigation of the interface between working memory and emotion, as well as hemispheric lateralization in emotion processing [26]. Our research using the EMOBACK task has previously demonstrated the importance of state-dependency for cognitive-emotional rTMS effects [25]. In fact, a large body of studies have shown that the effects of rTMS strongly depend on the level of neuronal activity at the time of stimulation, by using preceding manipulations such as the presentation of visual cues [27,28] or the application of transcranial direct current stimulation (tDCS) [25,29].

The current study is the first to investigate the state-dependent effects of rTMS on emotional working memory in healthy participants by using colored light exposure (blue versus green) prior and concomitant to prefrontal stimulation. We hypothesized that rTMS would augment task performance to a greater extent under blue light in comparison to green light exposure.

## 2. Materials and Methods

### 2.1. Sample

Twenty healthy volunteers (12 males, 26 ± 4 years) participated in this study after giving written informed consent. All subjects were native German speakers, had 12 or more years of formal education and normal or corrected-to-normal vision. Sixteen of the 20 participants completed a verbal intelligence test (Mehrfachwahl-Wortschatz-Intelligenztest, MWT-B, [30]) and presented an intelligence score within the normal range. They reported no concomitant neurological or psychiatric conditions or any contradictions to rTMS [31]. All participants were naïve to rTMS stimulation and received financial compensation for participation. The study was conducted in accordance with the Declaration of Helsinki and local ethics board approval (Charité Berlin, Germany, ethical approval number EA4/047/11).

### 2.2. Study Design

**Figure 1:**
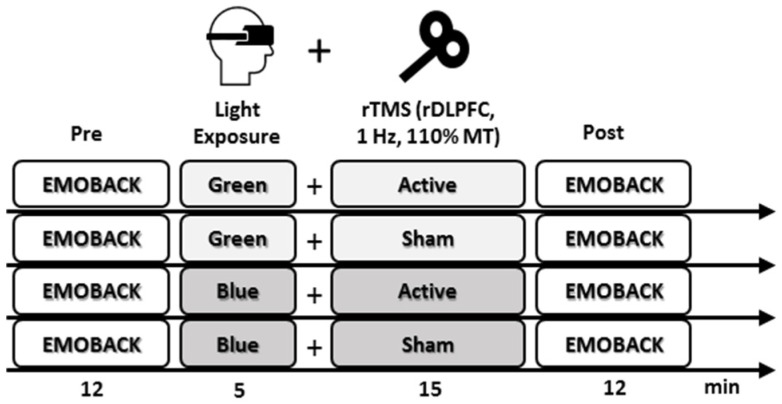
Schematic illustration of the experimental within-subjects design. Emotional n-back (EMOBACK) task performance was measured at baseline (Pre) and after light exposure (green/blue), combined with low-frequency repetitive transcranial magnetic stimulation (rTMS) (active/sham) applied over the right dorsolateral prefrontal cortex (DLPFC) (Post).

In a single-blind randomized within-subjects design, all participants underwent four experimental conditions; in each condition, they received active or sham rTMS over the right DLPFC in combination with blue or green light exposure. To counteract possible order effects, participants were randomly assigned to different orders of conditions (4 conditions result in 24 different orders) via a random number generator. Colors were presented via video glasses (Wrap 920, Vuzix, Rochester, NY, USA) 5 min prior and 15 min concomitant to active or sham rTMS. The video eyewear provides a 67-inch dual monitor display, as seen from 10 feet. In accordance with previous research using colored light exposure to study cognitive processing [11,12,13], participants were exposed to monochromatic illuminations at 470 nm (blue) and 550 nm (green). The brightness of both light exposures was computed to be identical, so that blue light stimulation of the intrinsically photosensitive retinal ganglion cells would be equal to the stimulation of the classical photoreception systems via green light. In each experimental condition, the EMOBACK task [24,25,26] and a preceding mood questionnaire [32] was completed pre- and post-intervention. The study was completed in two time-matched study sessions a week apart, each containing two experimental conditions (see Figure 1). Successive sessions on the same day were separated by a 45 min washout period to avoid carry-over effects [24,25,33,34].

### 2.3. EMOBACK Task

Participants performed a 3-back verbal emotional n-back (EMOBACK) task. More specifically, they viewed sequences of words and were instructed to press a button as quickly and accurately as possible whenever a presented word was the same as the one shown three trials earlier. Participants were instructed to press the button on a keyboard with the same finger (right index or middle finger). Intensive training of the task was conducted prior to the actual experiment. EMOBACK stimuli were presented in a randomized sequence and consisted of positive, negative, and neutral nouns [35]. Arousal was controlled between positive and negative condition (ts < 1). All three emotion conditions were matched on valence, arousal, imageability, frequency, and numbers of letters (Fs > 1) (see Table 1). Four parallel word sets were used for the experimental conditions (ts < 1). Words were presented as white uppercase letters in the center of a black screen for 500 ms with an interstimulus interval of 1500 ms. Eighteen words of the same emotion condition were presented in a block, followed by a 10–14 s fixation period. The total number of stimuli was 2160 (540 stimuli for each of the four experimental conditions). The EMOBACK task was programmed using Presentation software (Version 14.5, Neurobehavioral Systems Inc., San Francisco, CA, USA). In addition, after each experimental condition, participants were requested to perform valence and arousal ratings for all words presented in the EMOBACK task.

### 2.4. Mood Assessment

All participants completed the Multidimensional Mood Questionnaire (MDBF) [36] at baseline and immediately after experimental stimulation. The scale is comprised of 24 adjectives with a five-point rating scale, corresponding to three emotional dimensions: elevated–depressed mood, wakefulness–tiredness, and calmness–nervousness.

### 2.5. rTMS Application

We used a Medtronic stimulator (MagPro X100, MagVenture, Farum, Denmark) connected to a figure-of-eight coil (MCF-B65). Before rTMS application, the optimal stimulation site of the right DLPFC (Brodmann area 9/46) was marked individually using magnetic resonance imaging (MRI) non-stereotactic guidance [37]. Anatomical images were acquired with a Siemens Trio 3T scanner (176 T1-weighted images, 1 mm slices). Neuronavigation (eXimia, Nexstim, Helsinki, Finland) was used to position the coil perpendicular to the marked stimulation point on the skull at an angle of 45° with respect to the sagittal direction. The exact coil position was maintained with an adjustable arm throughout the experiment. A placebo coil (MCF-P-B65) was positioned in exactly the same manner for sham stimulation, which looks and sounds identical to the real coil and provides focal scalp electrical stimulation for a similar sensation. Participants received 15 min of 1Hz rTMS at 110% of the resting motor threshold, which was defined as the minimum intensity capable of evoking motor potentials of at least 50 µV recorded from the right first dorsal interosseus (FDI) in 5/10 stimulations. During stimulation, all subjects wore earplugs.

### 2.6. Statistical Analysis

Primary outcome measures were accuracy and reaction time in the EMOBACK task. Accuracy was defined as the ratio of correct responses (hits and correct rejections) to total number of stimuli. Mean reaction times of correct responses were additionally analyzed. To study stimulation effects, a four-way repeated measures ANOVA was applied to accuracy and reaction times with the factors: light exposure (blue, green), stimulation (active, sham), emotion (positive, negative, neutral) and time (pre, post). To evaluate changes in participants’ current mood states, all MDBF subscales were analyzed using repeated measures ANOVAs with the factors: light (blue, green), stimulation (active, sham), and time (pre, post). Data of one subject were lost due to technical problems. Furthermore, individual valence and arousal ratings of the stimulus material were analyzed using repeated measures ANOVAs with the factor of emotion (positive, negative, neutral) on each of the four sets. The Greenhouse–Geisser correction was used where applicable, and post hoc *t*-tests with Bonferroni correction were performed to characterize the significant effects. All tests were two-tailed, and the significant threshold was set at *p* < 0.05. Statistical analyses were carried out using SPSS Statistics (Version 25.0. Armonk, NY, USA: IBM Corp.).

## 3. Results

### 3.1. Emotional Working Memory

**Figure 2:**
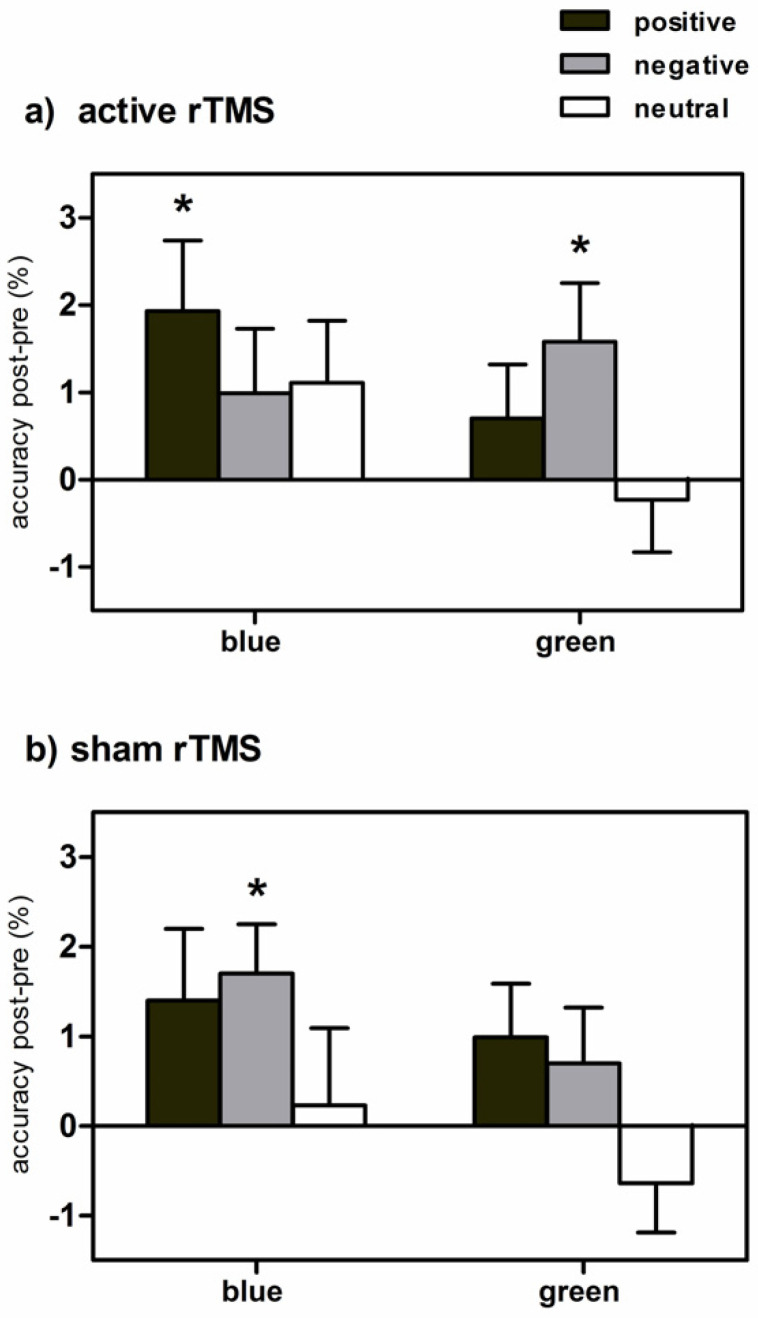
Valence-specific accuracy differences (post minus pre) in the EMOBACK task from baseline under blue and green light exposure after (**a**) active and (**b**) sham rTMS application over the right DLPFC. * *p* < 0.05).

The repeated measures ANOVA on accuracy revealed a significant emotion by time interaction (F(2,38) = 5.58, *p* < 0.01, η² = 0.23). Post hoc t-tests showed that participants performed less accurately for emotional as compared to neutral words at baseline (positive: t(1,19) = −2.62, *p* < 0.05, negative: t(1,19) = −2.35, *p* < 0.05). Interestingly, task accuracy increased significantly for emotional words across experimental conditions (positive: t(1,19) = −2.57, *p* < 0.05, negative: t(1,19) = −4.29, *p* < 0.001). Furthermore, we found a significant three-fold light exposure by stimulation by emotion interaction (F(2,38) = 3.31, *p* < 0.05, η² = 0.15), indicating the specific effects of experimental conditions on task accuracy. After active rTMS (Figure 2a), performance significantly improved for positive words under blue light exposure (t(1,19) = −2.37, *p* < 0.05) and for negative words under green light exposure (t(1,19) = −2.46, *p* < 0.05). After sham rTMS (Figure 2b), significantly enhanced performance was additionally found for negative words under blue light exposure (t(1,19) = −3.07, *p* < 0.01). No effects were found in the repeated measures ANOVA on reaction times. All accuracy and reaction time values can be found in Table 2.

### 3.2. Mood Assessment

With regard to potential mood effects of the intervention, we found a main effect of time in the wakefulness–sleepiness subscale (F(1,16) = 29.53, *p* < 0.001, η² = 0.65), indicating that participants felt generally more tired at the end of an experimental session. Table 3 provides details of all MDBF results.

### 3.3. Word Rating

Repeated measures ANOVAs on individual word rating on each of the four sets (Table 4) revealed a main effect of emotion on both arousal and valence on each of the four sets. Contrasts in every set showed that both positive and negative words were rated as more arousing than neutral words (*p* < 0.001), but not significantly different amongst these two (ts < 1). All three emotion categories were significantly different from each other in valence (*p* < 0.001).

## 4. Discussion

This study aimed to explore the state-dependent effects of light exposure combined with right prefrontal rTMS on performance in an emotional n-back (EMOBACK) task. By including emotion-laden words in the EMOBACK task, we were able to explore the interaction between working memory and emotion, as well as hemispheric lateralization in emotion processing. To our knowledge, the current study is the first to explore the light-dependent effects of rTMS on cognitive-emotional functions in healthy participants. In a randomized within-subjects design, 20 participants were exposed to four conditions, combining blue or green light with active or sham rTMS applied to the right DLPFC. In each condition, the EMOBACK task was presented pre- and post-intervention.

Independent of the effects of the applied intervention, we first found a detrimental effect of emotional content on working memory. At baseline, participants responded less accurately to both positive and negative words as compared to neutral words. This finding is in line with previous studies, including our own work [24,25,38,39,40]. It has been argued that emotionally salient stimuli capture and hold attention, resulting in increased task demands for higher executive functions such as working memory [39]. This is also in line with a recent meta-analysis studying the impact of affective information on working memory at a neural level. Results show that affective stimuli activate cerebral regions of the salience network (including the amygdala and hippocampus) as well as components of the executive frontoparietal control network [41].

Regarding the effects of the intervention, we found valence-specific rTMS effects in dependence of colored light exposure; task accuracy was significantly increased for positive words under blue light exposure and for negative words under green light exposure. Furthermore, blue light combined with sham rTMS showed improved performance for negative words (Figure 2). Importantly, these effects could not be attributed to changes in mood or alertness induced by the different experimental interventions. On a neural level, blue light exposure has been shown to activate brain networks that underlie cognitive performance, especially frontal and parietal areas associated with working memory [1,11,13,14]. Furthermore, blue light increased activity in the hippocampus and amygdala immediately after onset, suggesting that blue light might modulate emotional processing by limbic responses [1]. On a behavioral level, we found a significant increase in EMOBACK task performance, especially for positive words after blue light exposure combined with active low-frequency rTMS. A potential mechanism underlying these results might be seen as an inhibition of the right DLPFC, which is dominant in the processing of negative emotions according to the valence hypothesis [42].

Taken together, the applied stimulation may have reduced the attentional bias towards negative stimuli [43], resulting in enhanced task accuracy for positive stimuli, augmented by higher alertness and arousal processes through the blue enriched condition and its activation of the frontoparietal control network. In contrast, green light has been shown to reduce arousal responses [5] and to decrease brain activity during working memory tasks [1,13]. Consequently, it can be speculated that in the green light condition, the attention network of participants had been weakened through reduced arousal and alertness responses, opposing the stimulation effects of rTMS and generating a return to baseline cognitive levels, with negative stimuli attended preferentially through the right hemisphere [42]. This might explain the significant increase in EMOBACK task performance, especially for negative words in that condition. A stronger recruitment of the right DLPFC during processing of negative words in the EMOBACK task has been previously shown in our own work using the same task in combination with fMRI [26] and rTMS [24,25]. However, further assessment is needed to understand the underlying mechanisms of the combined intervention of rTMS and light exposure, and to address the functional relevance of prefrontal as well as limbic responses in this approach.

It should be noted that the analysis of task performance revealed a pronounced ceiling effect in our EMOBACK task across experimental conditions. Interestingly, previous research by Hoy and colleagues [44] found that intermittent Theta Burst Stimulation (iTBS) to the left DLPFC significantly enhanced task-related gamma power during a 3-back task, without showing a significant improvement on the behavioral level of task performance. The authors suggested a possible behavioral “enhancement ceiling effect”, especially during 3-back performance in healthy participants. Thus, significant neural changes in areas relevant for task processing could not alter the performance ceiling. Although we did find a small but significant improvement in our 3-back task despite a pronounced ceiling effect, the ability of our stimulation conditions to produce larger behavioral changes in working memory function may be limited. It can be speculated that our novel stimulation approach of combining light exposure and rTMS might have more relevance to the restoration of impaired cognitive functioning in clinical populations.

Furthermore, it is worth highlighting that we found no current mood effects of our intervention, as assessed with a mood questionnaire before and after the application of the combined intervention. Therefore, our results cannot be attributed to different emotional states. This is in line with previous research examining mood effects of one session of low-frequency prefrontal rTMS, causing no immediate mood changes in healthy volunteers [24,45,46]. In this context, it is worth highlighting that both rTMS [47] as well as light therapy [48] are established treatment methods for mood disorders including major depressive disorder. Interestingly, in a recent study, Mania and Kaur [49] introduced proof of concept for a novel promising antidepressant approach by combining light therapy and rTMS. Six patients with severe treatment-resistant depression received deep TMS to the DLPFC according to the FDA-approved protocol [50]. Light therapy was administered simultaneously for 20 min. All patients completed the combined treatment course without any side effects. The authors discuss that exposure to bright light can activate neural circuits involved in depression and, therefore, may have both synergistic and priming effects when combined with rTMS. In fact, combining both interventions in a clinical context is convenient, safe to use, and simple to set up and deliver. Whether this combined treatment approach shows a superior clinical outcome as compared to monotherapy using rTMS or light therapy is an important question for future work.

Word processing can be strongly influenced by the level of valence and arousal [51], which are supported by distinct neural systems [52,53]. Therefore, a strength of the present study lies in the carefully matched selection of words with equal valence and arousal levels for positive and negative words according to the Berlin Affective Word List (BAWL) norms [35]. We additionally controlled our four stimuli sets with regard to imaginability and frequency, as well as the number of letters and syllables whose relevance has been shown in tasks relying on word material [54]. To further control the stimulus material in our specific study sample, all participants were instructed to rate arousal and valence levels of all EMOBACK words at the end of the experiment. The results of these individual word ratings confirmed a successful matching of our stimuli sets according to the BAWL norms. Importantly, we also found no significant differences for arousal and valence ratings between the different stimulation conditions. This indicates that the reported effects of the intervention cannot be explained by the different emotional content of the four parallel word sets used for the experimental conditions.

Our findings of light-dependent rTMS responses, in combination with previous research regarding cognitive-emotional effects under blue light conditions [1,2,3,4,5,6], should be considered in future study designs using non-invasive brain stimulation. Natural daylight is on the blue end of the range of color temperatures, while an incandescent lightbulb emits lower warmer light [55]. Therefore, a study setting with natural blue daylight, which is only present during the strongest sun irradiation hours [55,56], could affect cognitive-emotional neuromodulation independent of the applied stimulation [57,58]. We thus recommend that future studies control light conditions during rTMS experiments.

There are several limitations to this study. First, our study sample was relatively small (N = 20) and results will benefit from further replication in larger studies as well as extension into clinical populations with impaired cognitive functioning. In addition, the use of techniques such as concurrent TMS-EEG or TMS-fMRI is required to better understand the neural mechanisms underlying the cognitive effects of the combined intervention of rTMS and colored light exposure. Second, our study was completed in two study sessions on different days (one week apart). However, each day contained two experimental conditions, separated by a washout period of 45 minutes to avoid carry-over effects [24,25,33,34]. Although it has been estimated that cognitive after-effects of rTMS studies using 1 Hz protocols last approximately as long as the duration of the stimulation itself (i.e., 15 min in our study design) [59], it is important to note that we cannot fully exclude carry-over effects of rTMS stimulation to the second experimental condition on the same day. Third, no control light (white) or the absence of light has been implemented as a condition in our study design. Future studies should investigate whether cognitive-emotional effects are different between wavelength exposures of specific colors as well as in comparison to an additional control condition. Finally, a number of additional factors might influence affective and cognitive reactions to specific light parameters (e.g., flickering rate [60,61]) as well as to non-invasive brain stimulation [62], and should be further explored in future research.

## 5. Conclusions

In conclusion, our study shows colored light exposure to be a potential adjunctive technique to non-invasive brain stimulation. Future studies are needed to thoroughly understand how blue light modulates the cognitive-emotional effects of rTMS and may help to improve state-dependent outcomes of rTMS in clinical treatments.

## Figures and Tables

**Table 1:** Stimulus characteristics used in the four parallel versions of the emotional n-back (EMOBACK) task.

		Set 1			Set 2			Set 3			Set 4	
	Positive	Negative	Neutral	Positive	Negative	Neutral	Positive	Negative	Neutral	Positive	Negative	Neutral
	M (SD)	M (SD)	M (SD)	M (SD)	M (SD)	M (SD)	M (SD)	M (SD)	M (SD)	M (SD)	M (SD)	M (SD)
Valence	1.9 (0.4)	−1.7 (0.4)	0.0 (0.1)	1.8 (0.5)	−1.9 (0.5)	0.0 (0.1)	1.8 (0.5)	−1.8 (0.6)	0.0 (0.1)	1.9 (0.3)	−1.8 (0.5)	0.0 (0.1)
Arousal	3.1 (0.5)	3.2 (0.6)	2.2 (0.2)	3.2 (0.6)	3.2 (0.6)	2.1 (0.3)	3.2 (0.6)	3.3 (0.6)	2.2 (0.3)	3.2 (0.5)	3.2 (0.5)	2.2 (0.2)
Imageability	4.1 (1.3)	4.4 (1.2)	4.1 (1.3)	4.3 (1.3)	3.9 (0.9)	4.1 (1.3)	4.5 (1.2)	4.3 (1.3)	4.0 (1.3)	4.1 (1.2)	4.3 (1.2)	3.9 (1.3)
Frequency	0.8 (0.9)	0.6 (0.8)	0.7 (0.6)	0.7 (0.7)	0.6 (0.9)	0.7 (0.8)	0.7 (0.7)	0.5 (0.7)	0.7 (0.8)	0.7 (0.7)	0.8 (0.7)	0.7 (0.7)
Letters	6.9 (1.3)	6.4 (1.4)	6.5 (1.3)	6.6 (1.4)	6.6 (1.6)	6.5 (1.4)	6.4 (1.5)	6.2 (1.4)	6.2 (1.3)	6.6 (1.4)	6.5 (1.5)	6.4 (1.3)
Syllables	2.2 (0.6)	2 (0.7)	2. (0.5)	2.4 (0.6)	2.1 (0.7)	2.3 (0.6)	2.2 (0.7)	1.8 (0.5)	2.2 (0.6)	2.1 (0.6)	2.3 (0.7)	2.3 (0.7)

Abbreviations: M = mean; SD = standard deviation.

**Table 2:** EMOBACK task performance separated for emotion conditions (positive, negative, neutral) at baseline (pre) and after stimulation (post).

			Pre			Post		
			Positive	Negative	Neutral	Positive	Negative	Neutral
			M (SD)	M (SD)	M (SD)	M (SD)	M (SD)	M (SD)
Accuracy (%)	Blue Light	Active rTMS	93.2 (5.5)	95.3 (4.0)	94.4 (3.5)	95 (3.9)	96.2 (3.2)	95.6 (3.1)
		Sham rTMS	94.8 (4.5)	94.7 (3.4)	95.9 (4.2)	96.2 (2.8)	96.3 (3)	95.6 (3.4)
	Green Light	Active rTMS	94.9 (3.8)	94.7 (3.5)	96.2 (2.7)	95.6 (3.3)	96.6 (2.9)	96.1 (3.2)
		Sham rTMS	94.7 (3.5)	95.5 (2.9)	96.3 (2.9)	95.6 (3.9)	96.2 (3.2)	95.5 (3.3)
Reaction Time (ms)	Blue Light	Active rTMS	692.0 (199.9)	703.7 (212.3)	651.4 (193.6)	684.7 (207.2)	674.2 (201.3)	718.4 (175)
		Sham rTMS	690.5 (179.4)	705.6 (209.6)	691.3 (217.1)	707.6 (244.4)	687.7 (231.9)	724 (242.4)
	Green Light	Active rTMS	663.3 (187.4)	664.8 (153.5)	664.2 (174.3)	662.1 (208.3)	655.9 (196.7)	650.7 (199.8)
		Sham rTMS	721.6 (219.5)	668.3 (184.8)	672.6 (169.7)	655.7 (181.1)	657.4 (175.4)	666.3 (185.2)

Abbreviations: M= mean; SD= standard deviation; ms= milliseconds.

**Table 3:** Mood assessment before (pre) and after (post) light exposure combined with rTMS.

		Elevated—Depressed Mood		Wakefulness—Sleepiness		Calmness—Restlessness	
		Pre	Post	Pre	Post	Pre	Post
		M (SD)	M (SD)	M (SD)	M (SD)	M (SD)	M (SD)
Blue Light	Active rTMS	17 (3.4)	16 (3.1)	15 (3.0)	13 (3.3)	18 (2.7)	16 (3.7)
	Sham rTMS	17 (2.7)	17 (2.4)	14 (4.0)	13 (2.9)	17 (2.9)	17 (2.5)
Green Light	Active rTMS	17 (2.7)	16 (2.6)	16 (2.5)	12 (3.3)	17 (2.2)	17 (3.6)
	Sham rTMS	17 (2.3)	17 (2.9)	16 (3.8)	11 (3.7)	17 (2.8)	17 (2.6)

Abbreviations: M = mean; SD = standard deviation.

**Table 4:** Subjective valence and arousal ratings for all four sets (parallel versions) of the EMOBACK task.

		Valence M (SD)	Arousal M (SD)
**Set 1**	Positive	1.7 (0.4)	2.9 (0.4)
	Negative	−1.4 (0.6)	2.7 (0.5)
	Neutral	0.1 (0.1)	1.6 (0.2)
**Set 2**	Positive	1.9 (0.4)	3.1 (0.4)
	Negative	−1.0 (0.4)	2.6 (0.5)
	Neutral	0.3 (0.2)	1.6 (0.2)
**Set 3**	Positive	1.6 (0.4)	3.0 (0.4)
	Negative	−1.5 (0.5)	2.7 (0.3)
	Neutral	0.1 (0.1)	1.7 (0.2)
**Set 4**	Positive	1.5 (0.4)	2.9 (0.4)
	Negative	−1.5 (0.5)	2.8 (0.4)
	Neutral	0.1 (0.2)	1.6 (0.2)

Abbreviations: M= mean; SD= standard deviation.
